# Embedded FBG Sensor Based Impact Identification of CFRP Using Ensemble Learning

**DOI:** 10.3390/s21041452

**Published:** 2021-02-19

**Authors:** Jun Li, Yinghong Yu, Xinlin Qing

**Affiliations:** School of Aerospace Engineering, Xiamen University, Xiamen 361102, China; lijun@stu.xmu.edu.cn (J.L.); yuyinghong@stu.xmu.edu.cn (Y.Y.)

**Keywords:** composite structures, FBG sensors, impact identification, ensemble learning, SVR, BP neural network

## Abstract

Impact brings great threat to the composite structures that are extensively used in an aircraft. Therefore, it is necessary to develop an accurate and reliable impact monitoring method. In this paper, fiber Bragg grating (FBG) sensors are embedded in unidirectional carbon fiber reinforced plastics (CFRPs) during the manufacturing process to monitor the strain that is related to the elastic modulus and the state of resin. After that, an advanced impact identification model is proposed. Support vector regression (SVR) and a back propagation (BP) neural network are combined appropriately in this stacking-based ensemble learning model. Then, the model is trained and tested through hundreds of impacts, and the corresponding strain responses are recorded by the embedded FBG sensors. Finally, the performances of different models are compared, and the influence of the time of arrival (ToA) on the neural network is also explored. The results show that compared with a single neural network, ensemble learning has a better capability in impact identification.

## 1. Introduction

Composite structures have been widely used in the aerospace field due to their excellent properties, including high specific stiffness, specific strength, and devisable material properties [1,2,3]. The properties of resin matrix composites largely depend on the curing process of the resin. If the information of temperature, stress, and strain can be collected during the curing process, real-time monitoring of the composite fabrication can be realized, which can help improve the performance of composite products [4,5,6]. For this reason, it is necessary to monitor the curing process of composite structures in real time. Various methods have been proposed to do this, including thermal methods [7], ultrasonic methods, [8] and electrical methods [9]. However, these methods not only have low precision and a high cost, but they can be only used for offline measurement of small products. Therefore, they cannot be widely used in practical production. For real-time strain measurement, many methods based on visual technique have been proposed [10,11,12]. Due to the accurate results and noncontact characteristics of these methods, they are widely used in the field of civil engineering and have made great achievements. However, these methods have not been used to monitor the curing process of composite structures.

With the increasing maturity of optical fiber sensing technology, the application of optical fiber sensors in the real-time monitoring of composite molding process has developed, including the influence of optical fiber on the mechanical properties of composite products, optical fiber embedding technology, extraction and evaluation of solidification information, etc. [13,14,15,16,17]. The results show that fiber Bragg grating (FBG) sensors can be embedded inside the composite laminates, and that they have almost no effect on the mechanical properties of the composite structures due to them being small in size and lightweight. Moreover, an optical signal is a type of transmission with high sensitivity and low loss, and because of the electrical insulation of an optical fiber sensor, it is not affected by electricity and magnetic interference. The transmission of an optical signal will not produce sparks, and so the optical fiber sensor can be used in flammable and explosive environments [18]. These characteristics provide the possibility for the real-time monitoring of composite structures in both manufacturing and service.

Composite structures could be easily damaged by low velocity impact, such as flying birds or falling stones. In these cases, cracking, debonding, and delamination could occur with a high probability, and although they are barely visible, they could lead to structural internal imperfection [19,20]. Therefore, the impact identification of composite structures is of great importance for aircraft design and service [20,21,22]. However, it is extremely difficult to directly measure the real-time impact load on the structures in most cases. In order to solve this problem, many technologies have been developed, including a three-dimensional (3D) reconstruction technique [23] and sensor network technology. However, due to the complex service conditions of the aircraft, the 3D modeling technology is not applicable. With sensor network technology, a suitable network of sensors is arranged on the surface of the structure to collect the strain information, which can be used to monitor the impact loads. This is a more efficient method than others because the dynamic responses can be easily measured. For the passive impact monitoring in structural health monitoring (SHM) techniques, many identification approaches have been published.

The most original method for time-domain load identification is the direct inverse method based on a transfer function. However, a transfer function is difficult to be calculated, and the noise of the signal is hard to eliminate in application. Moreover, the stability of this method is unreliable [24]. The use of regularization methods, which mainly include direct regularization and indirect regularization, is the most effective way to improve these issues. The most used direct regularization methods are the Tikhonov regularization methods [21,22,23,24,25,26,27] and the singular value decomposition (SVD) methods [28,29,30]. By comparing the generalized SVD method, the Tikhonov regularization method, and the truncated SVD method, Jacquelin [31] found that these methods have better robustness, and the convergence speeds are faster, but how to calculate the regularization parameters is still a difficult problem. Khoo [32] identified the impact load using a pseudoinverse method. However, the experimental results show that the location of the load is more difficult to be identified compared with the load values. Zheng [33] used the coherent analysis method to optimize the placement of the sensors, which effectively decreased the computational effort.

Most of the methods mentioned above are only applicable to metal structures. However, due to the demand of realizing a lightweight and high-performance design concept, a large number of nonlinear composite structures have been used on new aircraft, which has brought great difficulties for the existing identification methods. 

With the development of artificial intelligence and the need to obtain more accurate results, artificial neural networks, an information processing technology, have been widely used in impact location identification. Byeong-Wook Jang et al. [34] used multichannel FBG sensors, combined with the wavelet transform and neural networks, to identify the impact location and estimate whether there is damage in the composite laminate. Leclerc et al. [35] used neural networks to identify the impact of complex aviation structures and obtained good results, indicating that neural networks are also suitable for complex structures. Su Yongzhen et al. [36] proposed a two-step method for the impact location of composite structures based on acoustic emission and neural network technology. The experimental results show that the method is effective. Frieden et al. [37] measured the dynamic strains of the structure by FBG sensors; they then analyzed the location of the impact combined with the time of arrival (ToA) and pointed out that one of the reasons for the inaccurate location identification is the uncertainty of the ToA. Therefore, it is necessary to explore the influence of the ToA on the neural networks.

The curing process and impact identification of composite structures are two important parts of life-cycle monitoring. In this paper, the embedded FBG sensors are used to monitor the curing process of unidirectional carbon fiber reinforced plastics (CFRPs). Then, an ensemble learning model based on a back propagation (BP) neural network and support vector regression (SVR) is proposed. The impacts and their corresponding strain responses are used to train and test the model. The results show that ensemble learning can identify the impact accurately with fewer training samples. Finally, the influence of the ToA on the identification model in unidirectional CFRPs is explored. The results show that even if the ToA of the strain signals is difficult to obtain, it is still worthwhile to do so, as it can provide effective information for the location identification of the impact. 

## 2. Theoretical Principles

### 2.1. Principle of Resin Curing Monitoring

During the curing process, cross-linking reaction will occur, resulting in a great change of the states of the resin. At the first period, the resin is heated and flows easily. Then, the onset of the cure shrinkage is developed; this stage is called the rubbery state. Finally, the thermoset resin becomes rigid and highly cross-linked polymer solids, which is called glassy state. During this process, FBG sensors can be embedded in the prepregs to monitor the whole chemical reaction state.

**Figure 1:**
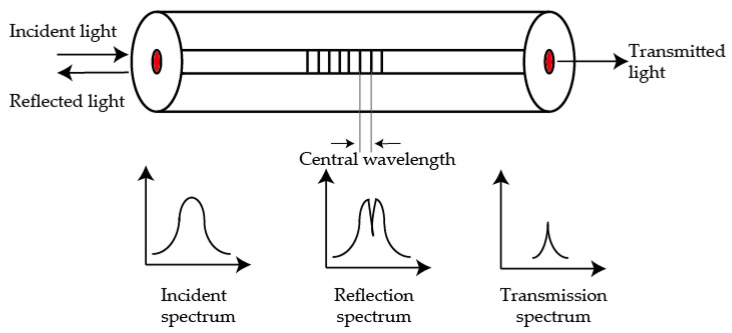
The working principle of a fiber Bragg grating (FBG) sensor.

The principle of FBG sensors is shown in Figure 1. When a beam of light enters the fiber, Bragg diffraction will occur at the grating with periodic spatial phase distribution. The incident light will be scattered out as a reflection spectrum.

The central wavelength $\kappa_{B}$ of the reflected spectrum satisfies Equation (1):

(1) $$\kappa_{B}=2n_{eff}\Lambda$$

where $n_{eff}$ is the effective refractive index and Λ is the period of refractive index change (grating period).

We differentiate both sides of Equation (1) to obtain

(2) $$d\kappa_{B}=2\Lambda \times dn_{eff}+2n_{eff}\times d\Lambda$$

Dividing both sides of Equation (2) by the center wavelength $\kappa_{B}$ results in

(3) $$\frac{d\kappa_{B}}{\kappa_{B}}=\frac{dn_{eff}}{n_{eff}}+\frac{d\Lambda }{\Lambda }$$

The first term on the right side of Equation (3) can be further transformed into

(4) $$\frac{dn_{eff}}{n_{eff}}=-\frac{n_{eff}{}^{2}}{2}[U_{12}-\nu (U_{11}+U_{12})]\varepsilon =(1-U)\varepsilon$$

where U is only related to the properties of the fiber and ε is the strain of the structure. Equation (4) can be expressed as a function of the strain and the center wavelength.

When the FBG sensor is embedded in the structure, the strain of the structure and chemical reaction will be reflected by the shift of the center wavelength. Therefore, the fiber demodulator can be used to monitor the strain of the curing process and the dynamic response under impact.

### 2.2. The Principle of Hilbert Transform (HT)

In application, due to the complexity of composite structures, there are usually noises in the strain responses collected by FBG sensors, which affects the calculation of the ToA and may lead to poor performance on the impact identification. 

**Figure 2:**
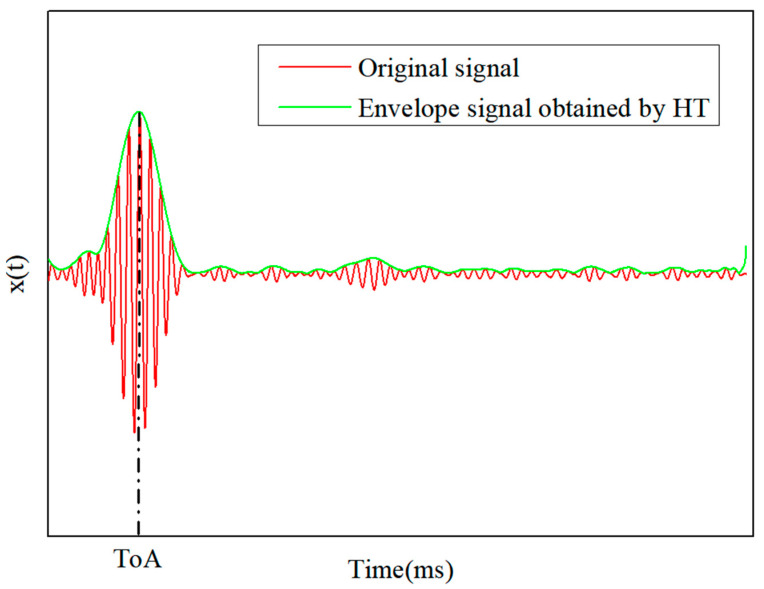
Schematic diagram of a Hilbert transform (HT) for the envelope signal.

Hilbert transform (HT) is a linear time-invariant filter used in signal processing [38]. HT is used to calculate the envelope signal of the collected strain responses. The abscissa corresponding to the maximum value of the envelope signal is usually used to calculate the ToA, and it can effectively distinguish the sequence of the ToA of each sensor. A schematic diagram of the envelope signal obtained by the HT is shown in Figure 2. The signal after the HT can be obtained by Equation (5).

(5) $$H[x(t)]=\tilde{x}(t)=\frac{1}{\pi }PV\int_{-\infty }^{+\infty }\frac{x(s)}{t-s}ds$$

where s is the variable of integration and PV stands for the Cauchy principal value of the integral.

### 2.3. Stacking-Based Ensemble Learning Method

Ensemble learning is developed from human civilization, namely from the observation that a consensus of a decision made by multiple experts is considered reasonable. This kind of idea is also applied in machine learning [39]. Generally, by mapping features from the training data to class labels or by predicting a variable, it is possible to train SVR, logistic regression (LR), BP neural network, and convolutional neural networks (CNNs) to complete the identification task. However, the performance of those base learners may deteriorate due to variance, noise, and deviation. 

To solve those problems, multiple trained submodels (here, they are called base learners) are used and combined to improve identification results, which is the concept of ensemble learning. In other words, ensemble learning can be regarded as a family of classification or regression algorithms to improve generalization ability and reduce errors caused by variance, noise, and bias [40]. Therefore, ensemble learning can be used for the impact identification of complex structures, such as composite structures. 

**Figure 3:**
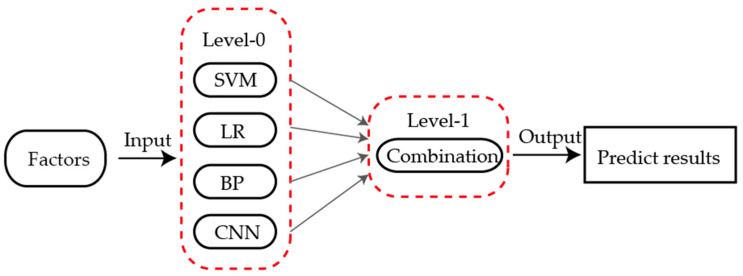
Schematic diagram of ensemble learning.

The stacking-based ensemble method was first proposed by Wolpert [41]. Unlike most existing ensemble learning methods, stacking uses meta-learner techniques to combine different types of algorithms. The structure of stacking consists of two levels, namely level-0 and level-1. The outputs of multiple base learners (level-0) are combined by the meta-learner (level-1), as shown in Figure 3 [42]. These machine learning methods from level-0 are commonly used for basic predictors, and the other methods from level-1 are usually for the combination of those base learners. 

**Figure 4:**
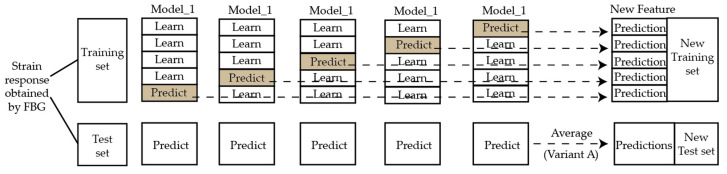
Stacking-based ensemble model for input data processing of level-0.

Typical combination methods include the weighted average and meta-learning method. By extracting information from the meta-data through stacking generalization technology, meta-learning has demonstrated satisfactory performance in many classification and regression tasks. Similarly, the stacking-based ensemble model was employed in this paper to obtain the ultimate ensemble forecasting results, as shown in Figure 4.

In the stacking-based ensemble learning method, when the impact occurs on the composite structures, the strain response could be recorded by FBG sensors. The impacts and their corresponding strain responses are generally divided into a training set and a test set. For a certain base learner model from level-0, cross validation is used on the training set, meaning that 80% of the data are used for training and 20% for testing. Then, the trained base learner model is also used to predict the test set. This process is repeated five times. The identification results from the training set is taken as a new training set, and the results from the test set are arithmetically averaged to get a new test set. The results of all base learners are combined as the input of level-1.

In the experiment, a base learner corresponds to one FBG sensor. The trained eight base learners were used independently to identify the impact by the strain responses recorded by the corresponding sensor, and then the preliminary identification results were set as the input of the meta-learner to predict the final results. Because of the similar types and parameters of sensors, the same neural network can be used as base learners to ensure the reliability of the identification results.

**Figure 5:**
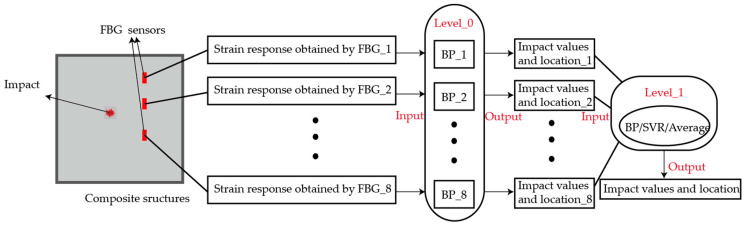
Schematic diagram of impact identification based on ensemble learning.

The weighted average method is the simplest combination method in ensemble learning. It is suitable for the samples that can be largely identified by the base learners. The BP neural network is one of the most widely used neural networks. The error back propagation algorithm is used to calculate parameters of the hidden layers systematically. Compared with other neural networks, the structure of a BP neural network is simple. In this paper, because the input and output of models are both low dimensions, BP neural networks can be used to get good results without increasing the computation. SVR is a new small sample learning method with solid theoretical foundation. It avoids the traditional process from induction to deduction and greatly simplifies the common classification and regression problems. The final results of SVR are only determined by a small number of support vectors, not the dimension of the sample space, which avoids the “dimensionality curse” to some extent. Therefore, in this paper, a BP neural network is used as the base learners, and three models (BP, SVR, and weighted average method) are selected as the meta-learner, as shown in Figure 5. 

#### 2.3.1. Weighted Average Method

Mean square error (MSE) is a measure of the difference between the estimator and the estimated. Here, t is defined as the mathematical expectation of ${\left(\theta -t\right)}^{2}$, which is an estimator of population parameter θ determined by samples. The mean square error of the estimator is then defined as $\sigma^{2}+b^{2}$, where $\sigma^{2}$ and b are the variance and bias of t, respectively. The MSE of the base learners in level-0 obtained during the training process is related to the weights in the meta-learning, as shown in Equation (6). The MSE greatly depends on the data type. Based on the data type in the experiment and the size of the specimen, we consider the model to have no identification ability when the MSE is above 10. When the MSE is less than 5, we consider the model to have good identification ability.

(6) $$\begin{array}{l} \mathrm{Impact}={\sum_{i=1}^{8}\lambda_{i}\cdot ({\mathrm{identification}}_{}\;results}_{}\;of\;base_{}\;learner\_i)\\ \lambda_{i}=1-\frac{base_{}\;learner\;\_i}{\sum_{i=1}^{8}MSE_{}\;of_{}\;(base_{}\;learner\;\_i)} \end{array}$$

#### 2.3.2. BP Neural Network and Bayesian Regularization 

The BP neural network is widely used in many applications due to its strong learning ability, fault tolerance, and induction ability. However, there are many limitations that exist, such as easily falling into the local minimum and overfitting during training. The Bayesian regularization algorithm is introduced to modify training performance and to improve the generalization ability of the model. The basic principle is detailed as follows.

The square error function (performance function) of the BP neural network is shown in Equation (7):

(7) $$E_{D}=\sum_{i=1}^{N}{\left(t_{i}-\phi_{i}\right)}^{2}$$

where N is the total number of samples, and $\phi_{i}$ and $t_{i}$ are the expected output and the actual output of the network trained for the first time, respectively. In order to improve the convergence speed and accuracy of the traditional algorithm, a penalty item is added to the Bayesian regularization optimization algorithm, and the training performance function is adjusted to Equation (8):

(8) $$F=\eta E_{w}+\mu E_{D}$$

where η and μ directly affect the training effect of the neural network. When both η and μ are small, the output of the training effect can be guaranteed to be smooth, and the possibility of overfitting can be reduced. Bayesian regularization adaptively adjusts the parameters of η and μ during the training process to make them optimal.

#### 2.3.3. Support Vector Regression Learning

Hinge loss function is used in support vector machines (SVMs) to calculate empirical risk, and a regularization term is added to the solution system to optimize structural risk. A SVM is a sparse and robust classifier. The principle of a SVM indicates that for a given training sample D, the function of the linear classifier is to find a hyperplane in the two-dimensional space based on D to separate the two types of samples. 

SVR was developed by Vapnik et al. [43] from SVMs. The basic principle of SVR is to find a hyperplane to minimize the distance between all given samples and the hyperplane. 

**Figure 6:**
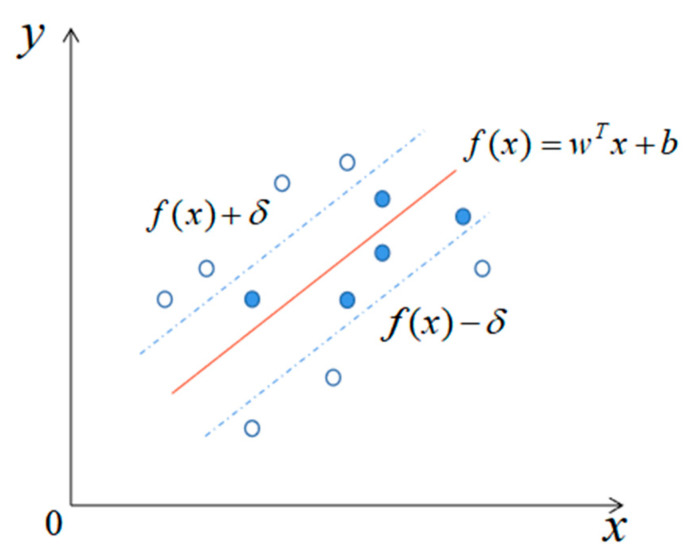
Schematic diagram of optimal hyperplane in support vector regression (SVR).

As shown in Figure 6, there are many such hyperplanes. The red line represents the hyperplane closest to all samples, which are also called support vectors. In SVR learning, as long as the deviation between the predicted value and the actual value is smaller than δ, the prediction is correct. The training problem of SVR is expressed by Equation (9):

(9) $$\begin{array}{l} \max_{}\;{}_{}L_{c}(\alpha_{i})=\frac{1}{2}\sum_{i,j=1}^{N}K(x_{i},x_{j}){\bar{\alpha }}_{i}{\bar{\alpha }}_{j}+\varepsilon \sum_{i=1}^{N}{\bar{\alpha }}_{i}-\sum_{i=1}^{N}y_{i}{\bar{\alpha }}_{i}\\ s.t._{{}_{}}\sum_{j=1}^{N}{\bar{\alpha }}_{i}=0{,}_{}{}_{}0\leq \alpha_{i}{,}_{}\alpha_{i}{}^{*}\leq C \end{array}$$

where C is a regularization parameter that penalizes data points containing errors greater than ε by a positive constant; ε is an insensitive parameter that defines a tube around the curve to be approximated; and ${\bar{\alpha }}_{i}$ are the decision variables, i.e., ${\bar{\alpha }}_{i}=\alpha_{i}-\alpha_{i}^{*}$, where $\alpha_{i}$ and $\alpha_{i}^{*}$ are the Lagrange multipliers. N is the training set cardinality, and $K(x_{i},x_{j})$ is the kernel function that could map vectors into a feature space to solve the regression problem of multidimensional data. The mathematical models for the SVR are given by Equation (10):

(10) $$f(x)=\sum_{i=1}^{\#SV}{\bar{\alpha }}_{i}^{*}K(x_{i},x_{j})+b$$

where #SV is the cardinality of the SV set, $\alpha_{i}^{*}$ and ${\bar{\alpha }}_{i}^{*}$ are the optimal values that satisfy Equation (10), and b is the bias that can be evaluated from the optimal alphas.

## 3. Experimental Setup

### 3.1. Experimental Setup for Cure Monitoring

**Figure 7:**
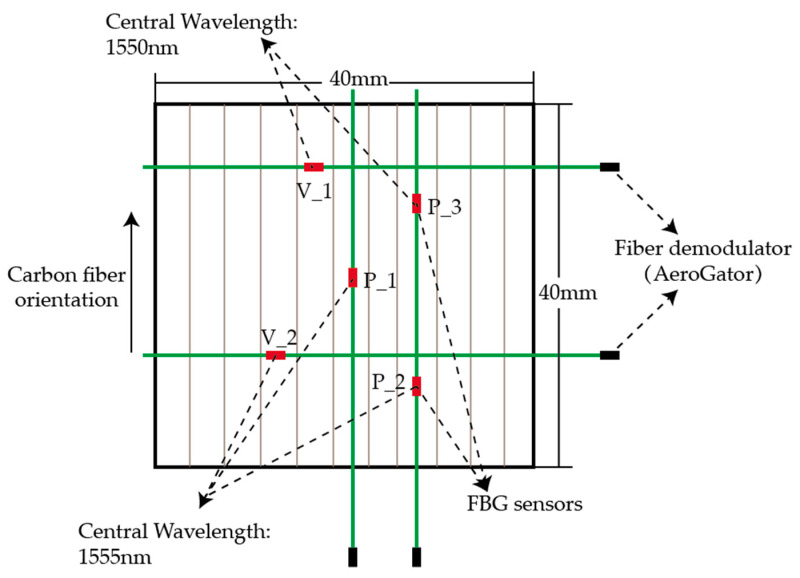
Layout of the FBG sensor in two orientations.

During the curing experiment, due to the anisotropy of CFRP, whether the angles between the fiber orientation and the FBG sensors affect the measurement results needs to be further explored. The laminate (400 mm × 400 mm × 1.835 mm), which consisted of eight unidirectional T300 prepregs, was placed in the central area of a 6061 aluminum alloy plate mold (600 mm × 600 mm × 2 mm). Five FBG sensors with same central wavelengths were embedded in the prepregs, with three of them along the carbon fiber orientation and other two in vertical fiber orientation, as shown in Figure 7. The vacuum bag molding method was applied in this experiment. AeroGator from Beijing Tongwei Technology Co. Ltd. was used as the fiber demodulator in the experiment. The comparison results are shown in Section 4. 

The results of the last experiment show that during the constant temperature stage, the angle between the sensors and the fiber orientations does not affect the monitoring results. Based on this conclusion, eight FBG sensors are embedded for the curing process monitoring. 

**Figure 8:**
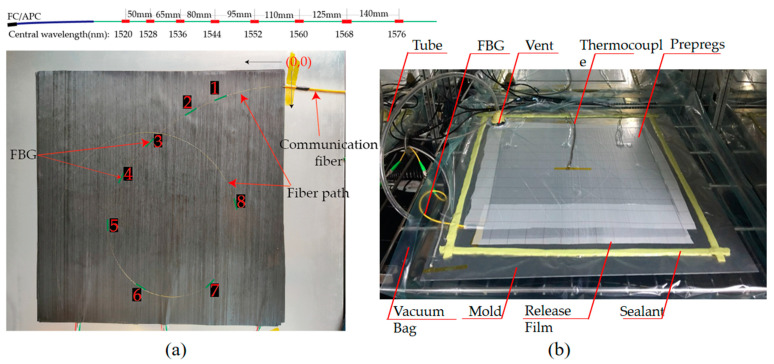
(**a**) Specifications and experimental layout of FBG sensors; (**b**) the scene picture of the experiment for the manufacturing process.

Due to the anisotropy of the composite plate, the propagation of the wave in the plate is complex. Frieden et al. [33] pointed out that the uncertainty of the ToA can affect the accuracy of impact identification. Theoretically, the ToA obtained by different sensors is easier to distinguish as the distances between the sensors increase. In order to explore how separating the sensors influence the accuracy of impact identification, the eight FBG sensors were first placed closely in the layout and then sparsely, as shown in Figure 8a. In the experiment, the eight sensors with different central wavelengths were connected in series. Due to the fragility of the optical fiber, there should not be small curvature points on the optical fiber path, which means that a rectangular layout scheme is not suitable. Meanwhile, for the same monitoring area, the fiber laid in a circular pattern can effectively reduce the length of the fiber embedded in the composite structure, which minimizes the effect of the sensors on the mechanical properties of the composite products. 

During the experiment, the mold was sealed and the air was pumped. Two thermocouples were placed on the corner of the aluminum tool plate and on the top of the vacuum bag, respectively. According to the resin cure curve, the whole system was cured at a constant temperature (105 degrees centigrade) for 3 h, and the strain was recorded by the FBG sensors every 3 min during the process, as shown in Figure 8b.

### 3.2. Experimental Instruments for Impact Identification

**Figure 9:**
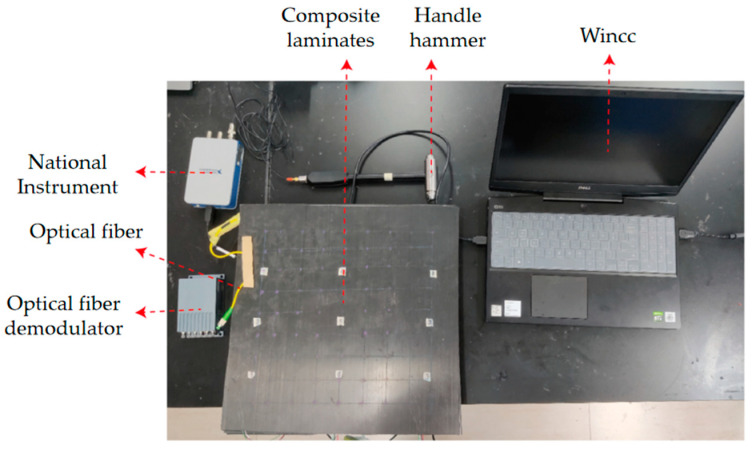
Construction of the experimental system.

After curing, the specimen was demolded, and the impact identification experiment was carried out in room temperature. In this experiment, the composite laminate was fixed on the table with a sealant to reduce the influence of echo wave. The handled hammer was used to generate the impact signal on the specimen. The signal can be recorded using National Instrument, which was connected to the hammer. Meanwhile, the demodulator was connected with FBG sensors to record the corresponding strain signals, as shown in Figure 9. 

### 3.3. Experimental Operation for Impact Identification

**Figure 10:**
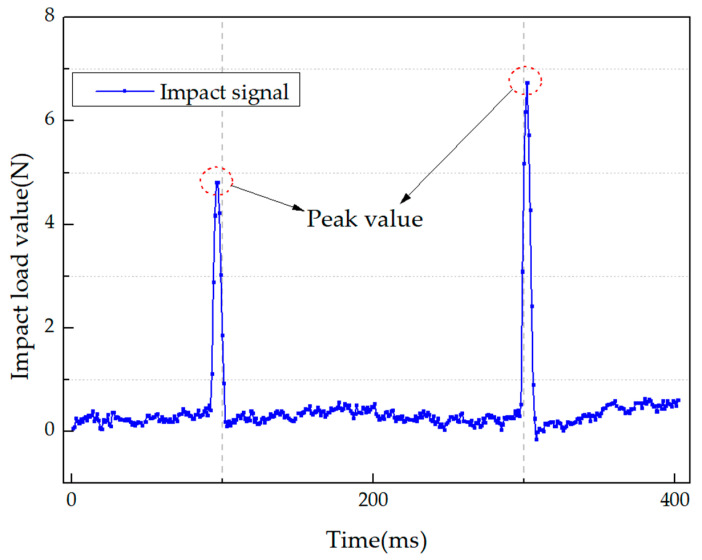
The peak value of impact signal of two successive impacts.

The whole experimental operation can be referred to in Figure 5. In the impact monitoring experiment, the specimen was firstly divided into 40 mm × 40 mm grids. Then, the hammer was used to create impact in each grid. The location of the impact was recorded manually, and the signal generated by the impact was transmitted through the pressure sensor on the hammer and recorded using National Instrument. As shown in Figure 10, the maximum value of the impact signal in this figure was defined as the peak value. At the same time, the strain responses caused by the impact were received by the FBG sensors embedded in the specimen and recorded by the fiber demodulator. The sampling frequency is 19 khz. After that, all the strain responses were processed by the HT. The envelope signals obtained by the HT were set as the input of the base learners. Meanwhile, the impact peak values and locations were set as the output of the base learners for training and testing. Finally, the preliminary identification results obtained by the base learners were set as the input of the meta-learner to predict the final results. A total of 295 groups of signals were collected on the specimen.

The process of establishing the model is the process of determining the parameters of the model. A variety of parameters and optimization algorithms were tried during the training process, and a group of parameters that minimize the MSE of the base learners was selected, as shown in Table 1. The neural networks were built by commercial software, and the identification results were obtained. 

## 4. Results and Discussion

### 4.1. Results and Discussion of Curing Process Monitoring

**Figure 11:**
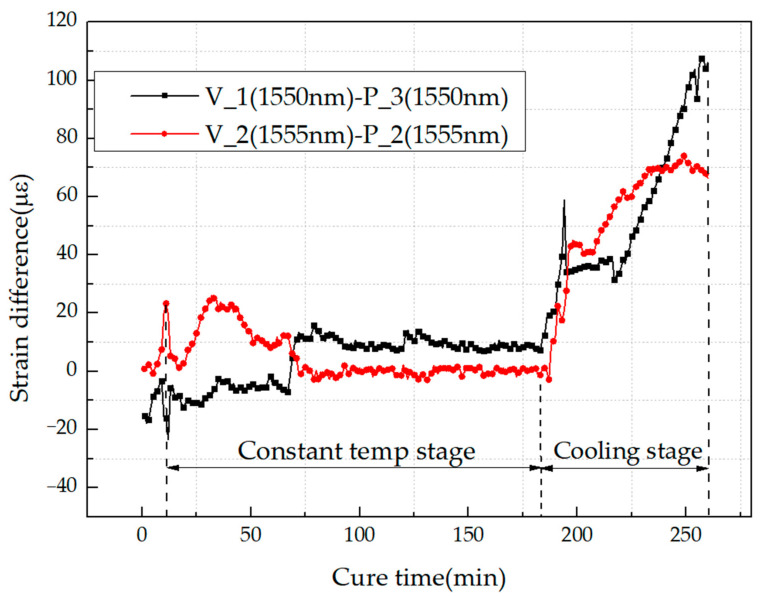
Differences of strain measured by FBG sensors in two orientations during the constant temperature stage and cooling stage.

The curing process mainly includes three stages: a heating stage, a constant temperature stage, and a cooling stage. Among them, the heating stage is very short, i.e., within 10 min. During this stage, the resin starts to change from the semicured state to the viscous flow state, which is the early stage before a cross-linking reaction. Therefore, only the strains of the other two stages were compared. FBG sensors with same wavelength have the same temperature coefficients, which means when the temperature changes, the strains measured by these FBG sensors are the same. However, for the FBG sensors with different central wavelengths, the strains will be different. In order to make the results comparable, the strains obtained by FBG sensors from two orientations were extracted. Among them, the differences of the strains measured by FBG sensors with the same wavelength were compared, as shown in Figure 11.

In the constant temperature stage, the strain change is mainly caused by the curing shrinkage of the resin. It can be seen from the Figure 11 that the difference of the strain measured by FBG sensors in two orientations was maintained within 50 in this stage, with no obvious difference. However, in the cooling stage, the strain was mainly affected by the thermal effect of the specimen, and so the strains from the two orientations became gradually different. 

**Figure 12:**
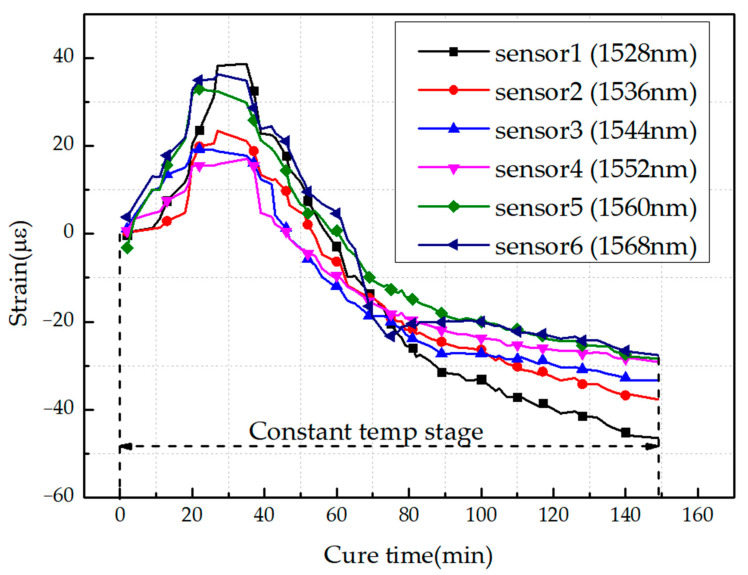
Strain of FBG sensors with different wavelengths during the constant temperature stage.

We assume that the angle between the sensors and fiber orientation will not affect the monitoring results when FBG sensors are used to monitor the strain of resin in the constant temperature stage. Moreover, the focus of this paper is to monitor the states of resin, which will be completed before the cooling stage. In addition, due to the particularity of the unidirectional composite sheet, there will be no obvious residual strain in the cooling stage. On account of the above reasons, in the curing monitoring process, only the strains in the constant temperature stage were analyzed. Some typical results during this stage are shown in Figure 12.

As we can see, in the first period (before 25 min), the epoxy resin is heated and flows easily, thereby reducing its viscosity, which is the main cause for the increase of strain. Then, the onset of the cure shrinkage develops at 30 min, and this time point is determined as the gel point. After the gel point, the resin begins to change from a viscous flow state to a high elastic state, causing the modulus of the resin to begin developing and increasing, which then causes the strains to begin decreasing. After 150 min, the strain tends to be stable, which means curing is basically completed at this moment.

It can be concluded that FBG sensors can be used to monitor the curing degree and the state change of resin in the constant temperature stage.

### 4.2. Results and Discussion of Impact Identification

Firstly, in order to preliminarily analyze the identification results of the basic learners and the BP neural network, the envelope signal was directly set as the input of the BP neural network for training and testing. Meanwhile, the envelope signal was set as the input of the base learners in level-0 of the ensemble learning model. The output includes two parts, i.e., the impact location and the peak value of the impact signal. The MSE was used to characterize the accuracy of the model. The results are shown in Table 2. 

As we can see, when the strain responses were directly set as the input of the BP neural network, there are too many strain responses in series and there is more noise, both of which affect the feature extraction of the neural network, leading to poor performance in the location and peak value identification. As for the result of the base learners, although there is relatively less noise for the strain signal of a single sensor, the impact information is also reduced, resulting in a large identification error of the base learners, especially in location identification. 

**Figure 13:**
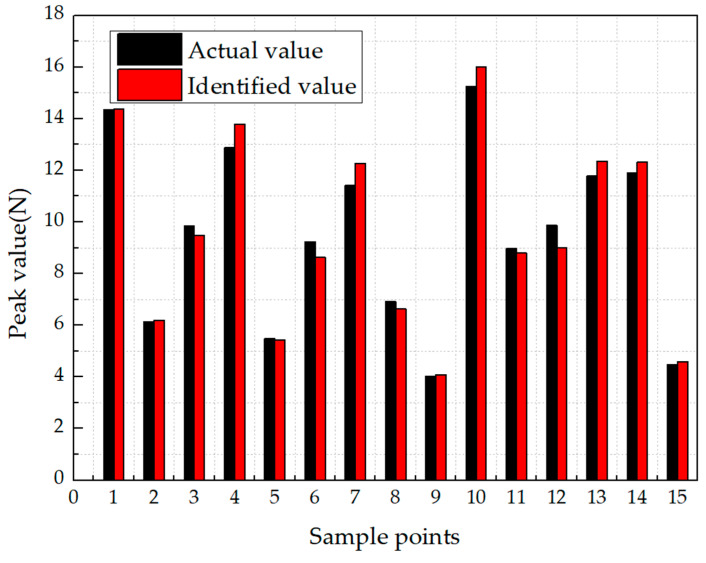
Identification results of peak values using ensemble learning.

However, compared with location identification, the identification results of the peak values are more accurate. The main cause of the result is that the peak value of the impact is mainly related to the waveform of the strain signal. Therefore, for the peak value identification, the combination method in level-1 was set to the weighted average to reduce the computation, as shown in Equation (6). The results are shown and compared in Figure 13 and Table 3.

In the identification of peak values, even if the same neural network was used, the MSE of the model can be effectively reduced from 3.23 to 1.12 by using ensemble learning. It can be seen from the Figure 13 that in most cases, the identified value was close to the actual value, and the accuracy of identification within 1N reaches 95.43%.

**Figure 14:**
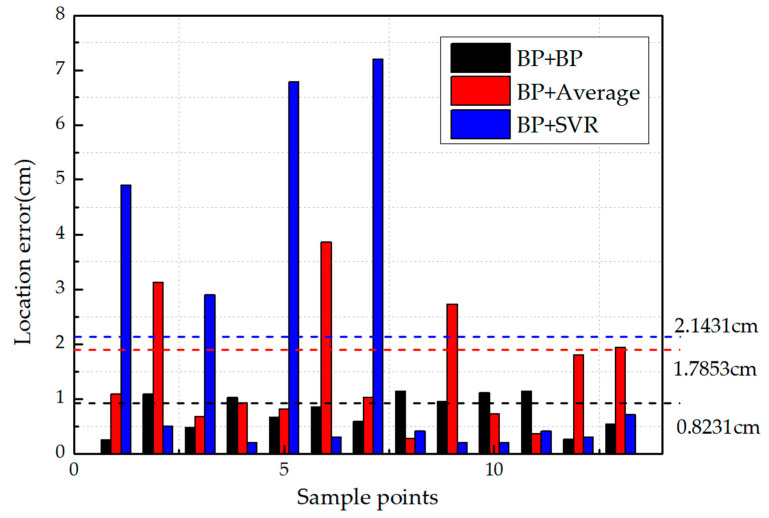
Comparison of location identification results of three models based on some sample points.

In the identification of impact location, because of the anisotropy of composite structures, the results of base learners in level-0 are poor during the training process. A simple weighted average cannot get accurate results. Therefore, SVR and BP neural networks are respectively used as a combination method in level-1. The results of level-0 were set as the input of the level-1 to obtain the final results, as shown in Figure 14. In order to further verify the performance of the different models in location identification, the goodness of fit ($R_{\nu }$) and the root mean square error (RMSE) of different models are calculated by Equations (11) and (12). The results are shown in Table 4.

(11) $$\begin{array}{l} Q=\sum_{i=1}^{N}{\left(y-f(x)\right)}^{2}\\ R_{\nu }=1-\sqrt{\left(\frac{Q}{\sum_{i=1}^{N}y^{2}}\right)} \end{array}$$

(12) $$RMSE=\sqrt{\frac{1}{N}\sum_{i=1}^{N}{\left(y-f(x)\right)}^{2}}$$

where N is the number of samples, and f(x) and y are the output of neural network and the actual measurement value, respectively. 

As we can see, compared with the BP neural network, the three models based on ensemble learning have better performance in impact location identification. When the combination method of level-1 was set to the weighted average, the average error is 1.7853 cm, and the error of some points is more than 3 cm. When the BP neural network was used as the combination method, the performance of the model is better, as shown in Table 4. 

When SVR was set as the level-1, except for the large error at some points, the identification error is less than 0.5 cm in most cases. The experimental results show that when the impact occurred at the edge of the specimen, the boundary echo is serious, which has a large effect on the signal. In this case, this model performed poorly, resulting in the average error of this model being 2.1431 cm. It can be concluded that using the BP (level-0) + SVR (level-1) model for ensemble learning has higher accuracy, but there is still a large error when the impact occurs at the edge of the specimen. This model has better performance based on the signal with a high signal to noise ratio (SNR). In contrast, the BP (level-0) + BP (level-1) model has better robustness. 

**Figure 15:**
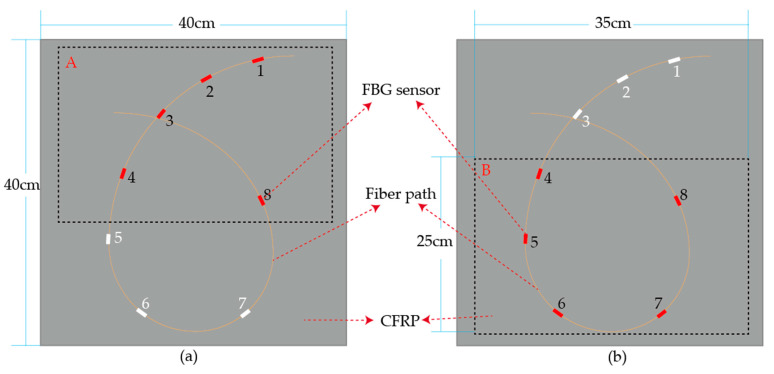
(**a**) Five FBG sensors with a close arrangement in group A; (**b**) five FBG sensors with a spaced-out arrangement in group B.

In order to explore the influence of sensor placement and distance on the accuracy of impact identification, two parts of the specimen with the same area are selected and were named group A and group B, respectively. Each part contains five FBG sensors. The arrangement of the five FBG sensors in group B is more spaced out than that in group A, as shown in Figure 15. 

**Figure 16:**
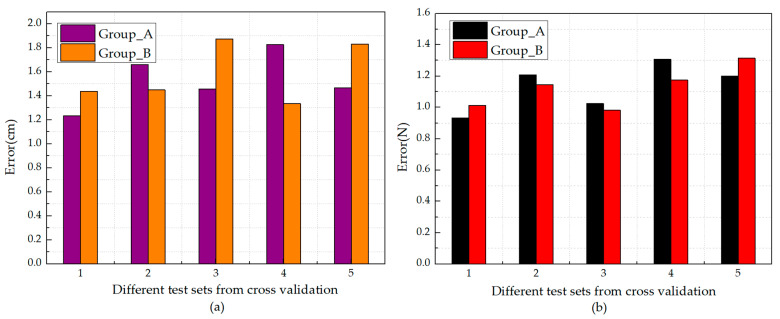
(**a**) Average error of location identification of impact based on five different test sets; (**b**) average error of peak value identification of impact based on the five different test sets.

The BP (level-0) + BP (level-1) model was used to identify the impact based on the impact signal and their corresponding strains measured by the selected five FBG sensors. Cross validation was used five times, and five different test sets were obtained. Each test set contained 30 groups of data that did not participate in the training process. The results of impact identification based on those five test sets were obtained, and the average error was calculated, as shown in Figure 16.

Figure 15 shows that there is no obvious difference between the results of group A and group B. It can be concluded that when the area and the number of sensors are the same, the space between the FBG sensors has no obvious effect on the performance of ensemble learning model in impact identification.

**Figure 17:**
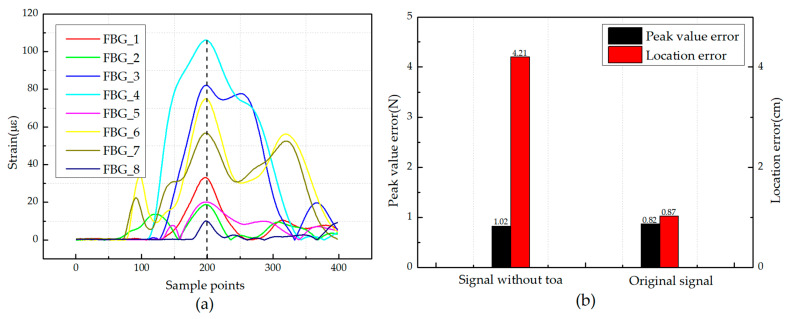
(**a**) Strain responses of different FBG sensors without the information of ToA; (**b**) comparison of impact identification results based on data without ToA and basic data.

Due to the anisotropy of the composite structures, the propagation of the wave in the CFRP is complex. It is necessary to study whether the ToA can provide effective information for the ensemble learning model. The abscissa corresponding to the maximum value of the envelope signal is usually used to calculate the ToA of each sensor. In order to eliminate the sequence of ToA in different signals, 200 strain data before and after the peak point of each signal were taken, meaning that the peak points were in the same position in the whole input signal. Then, the signal containing 400 strain data was set as the input of the BP-BP ensemble learning model. It can be considered that the new signal contains less information of the ToA compared with the original signals, as shown in Figure 17a. Meanwhile, the original signals from the same test set were set as the input of the same model for a control experiment. The results are compared and shown in Figure 17b.

The results show that although the ToA is difficult to obtain in the unidirectional CFRP, it can still provide effective information for the ensemble learning model. In addition, it can be concluded that the ToA mainly affects the location identification of the impact, but it has little effect on the peak value identification of the impact.

## 5. Conclusions

In order to improve the accuracy of impact identification and to monitor the curing process, FBG sensors were embedded in the prepregs during the manufacturing process. As an advanced type of sensor, FBG sensors can be used to monitor the curing degree of the resin matrix and the change of the elastic modulus in the constant temperature stage. Meanwhile, they can also produce a more sensitive response to the impact load in the service stage.

By monitoring the manufacturing process of unidirectional CFRPs, the degree of cross-linking reaction of composite structures can be clearly observed from a viscous flow state to a rubbery state and finally to the glassy state. This provides an extremely important basis for achieving high quality composite products. However, this monitoring scheme is only applicable to the constant temperature stage. In order to monitor the strain of CFRPs in the cooling stage, unbounded sensors are necessary, meaning more impurities should be added into the structure. Whether this solution will influence the monitoring of composite structures in service remains a topic of exploration for further studies.

A stacking-based ensemble learning model was also proposed in this paper for impact identification on composite unidirectional CFRPs. The results show that the trained ensemble learning model has better identification results than the BP neural network. Through the identification results of different ensemble learning models, it can be concluded that SVR has good identification ability based on the signals with high SNR, but the robustness of this model is poor. Finally, the strain responses without ToA information were set as the input of the ensemble learning model for impact identification. The comparison results show that even though the ToA is difficult to obtain, it can provide crucial information for the ensemble learning model to improve the accuracy of location identification. As a new model for impact identification on the composite sheet, ensemble learning provides the possibility for the online monitoring of aircraft in service. However, whether it is suitable for more complex structures remains to be further verified.

## Figures and Tables

**Table 1:** Parameters setting of base learners.

	Number of Hidden Layers	Number of Hidden Layer Nodes	Activation Function	Number of Iterations
BP	5	25	Sigmoid	500

**Table 2:** Comparison of the MSE between BP and the base learners in ensemble learning.

	BP	Base Learners in Ensemble Learning
MSE of peak value identification	3.23	FBG_1 = 4.33, FBG_2 = 6.24, FBG_3 = 5.58, FBG_4 = 4.89, FBG_5 = 5.47, FBG_6 = 5.84, FBG_7 = 6.13, FBG_8 = 7.41,
MSE of location identification	10.73	FBG_1 = 13.23, FBG_2 = 12.42, FBG_3 = 16.83, FBG_4 = 15.43, FBG_5 = 18.73, FBG_6 = 12.22, FBG_7 = 11.84, FBG_8 = 14.91,

**Table 3:** Comparison of peak value identification results in BP and ensemble learning.

	BP	BP (Level-0) + Average (Level-1)
MSE	3.23	1.12
Accuracy (within 1N)	62.41%	95.43%
Average error (N)	1.74	0.87

**Table 4:** Comparison of location identification results of three models based on four measurement indicators.

		Average Error (cm)	MSE	$R_{\nu }$	RMSE
BP		4.231	10.73	0.72	4.35
	BP (level-0) + Average (level-1)	1.785	2.51	0.88	2.24
Ensemble learning	BP (level-0) + SVR (level-1)	2.143	3.44	0.81	3.28
	BP (level-0) + BP (level-1)	0.823	1.21	0.94	1.37

## Data Availability

The data presented in this study are available in the article.
